# What Is the Best Method for Teaching Children to Care for Their Teeth?

**Published:** 1897-12

**Authors:** S. B. Hartman

**Affiliations:** Fort Wayne, Ind.


### What is the Best Method for Teaching Children to Care for their Teeth?

BY S. B. HARTMAN, D.D.S., FORT WAYNE, IND.

Read before the Bi-State Dental Meeting, South-Western Michigan, and Northern Indiana Societies, at Benton Harbor, Michigan, September 15th, 1897.

One of the most promising signs of the times, in the way of general knowledge of science and art by the people of the future, is that the day has dawned when no longer a slight understanding of the fundamental principles of education are sufficient to meet the requirements of a business or professional life, and the manner of teaching the young of to-day is far different than in the past. The great discoveries of the present time are the results of a greater reasoning power. The scholar to-day in our schools is farther advanced along the line of a general and broad education at fourteen or fifteen years of age than persons of eighteen or twenty, two decades in the past. This change has come from the tendency to reach out and grasp, analyze and study those things that are so closely connected with our well being, and from which we derive health and happiness. One cause that has produced this advancement has been the strengthening of reasoning powers through the use of illustrations and mechanical and chemical experiments. The child of to-day is taught cause and effect, and is no longer dwarfed with baby talk and kept in check in mental growth until he verges on manhood. Not only are his studies in the primary and preparatory departments of school life—those relating to such branches that may in future days be useful to him in business pursuits—but he is instructed to care for his body that it may be strong and healthy, that the great system of organs may perform their functions in harmony, what foods are most suitable for persons performing certain work, or those living in different localities. A chemical understanding of foods which will best promote growth, and most readily assimilate and replace waste and realize that one of the greatest studies of man is man.

The study of human physiology is no longer tedious and dry to the students in many of our public schools, for the methods of its teaching have changed, the text-books in this branch is not considered as a reader making little difference if what is read is comprehended or not because the teacher is unable to explain its teaching; but at this time, in many schools, local physicians frequently talk to the pupils on the relation that one organ sustains to another, its development, and how best to care for the same. They are taught the effect of medicines in assisting nature to cure disease or prevent it.

A gentleman in an eastern city related this occurrence to a friend: "Let me tell you what occurred at my table; a guest was taken dangerously ill at dinner—insensible—and there was a call for brandy to restore him; my little boy at once exclaimed, 'No, that is just what he don't need, it will paralyze the nerves and muscles of the blood-vessels so that they will not send back the blood to the heart.'

"When the liquor was poured out to give the man, the lad insisted on pushing it back, 'You will kill him, he has too much blood in his head already.'

"How did you know all that," his father asked.

"Why it is in my physiology at school."

It seems the text-books prepared by such men as Prof. Newell Martin, F. R. S. of Johns Hopkins University, had succeeded in giving the lad some definite information which was proving useful.

Thus we can observe how early impressions are retained in the mind and at an important moment manifest its power for good. Our profession, as a branch of the healing art, realize the great amount of health to the child if he can fully understand the importance of the teeth, the care they should receive, and when erupted, the diseases to which they are subjected, and what treatment should be given for their preservation.

Parents do not often comprehend the importance of caring for their children's teeth, although every other comfort, it seems a delight for them to grant, but are very indifferent to the teeth, yet, if the child could have some information and advice pertaining to his teeth, the parent would often be reminded by the child in reference to those organs so essential to digestion and facial expression.

The dental profession has long known the need of some method for the promotion of general information in reference to the teeth and associate parts of the mouth, and have resorted to various methods, more or less successful, such as newspaper articles, pamphlets, etc., yet the desired results are not accomplished, and to me the only way that will give the greatest fruits for the labor is talks personally to children. It has been my privilege on several occasions to give talks of this kind to children in the city of Fort Wayne, and it is surprising how much interest they manifest in the subject. If in every town or city, dentists would devote an hour or two in this manner, many a child would appreciate his efforts, and would be thankful to him.

Several years ago, at my first talk on "teeth" to school children, was present a little boy who carries newspapers out of school hours to aid in his support, and he has become so interested in caring for his teeth that now his visits to the dentist are frequent, and recently remarked, "I never want any of my teeth pulled," and this is not the only case where an appreciation has been shown. I have watched closely what results would follow, and a number of dentists have had calls from young persons in consequence. Perhaps you may wish to know how I conduct these school talks, and with the hope that when you return to your homes you may do likewise, or a better way, I relate my method of conducting them.

For about ten minutes at the commencement or introduction, my remarks pertain to digestion, and how the teeth prepare food for the same. Following, reference is made to facial expression, using charts to illustrate the teeth in irregular positions, and the unnatural appearance thus produced. By this time I have the attention of those I am addressing. I then talk on the deciduous teeth, time of eruption and shedding. I exhibit a number of specimens, showing conditions of teeth, lost prematurely, impressing upon the pupils the importance of keeping these organs until nature indicates the necessity of their removal, explaining what such indications are, exhibiting to them charts explaining the relation between temporary and permanent teeth, and why temporary teeth should be filled and receive care. After my remarks an opportunity is given for questions, and I have a general conversation on the teeth. I am pleased at this time to show you the charts I use on such occasions.

(REMARKS.—The essayist exhibited to the Association a number of charts, nicely prepared, which he has used at his talks on the teeth to school children in Fort Wayne, Indiana.)

